# From Stupor to Storm: A Diagnostic Dilemma of Neuroleptic Malignant Syndrome versus Malignant Catatonia

**DOI:** 10.1192/j.eurpsy.2026.12022

**Published:** 2026-07-31

**Authors:** Y. Aun, L. A. Ong, G. C. R. Tze - Lee

**Affiliations:** 1 Changi General Hospital, Changi; 2 Ng Teng Fong General Hospital, Jurong, Singapore

## Abstract

**Introduction:**

Neuroleptic malignant syndrome (NMS) is a rare, life-threatening emergency defined by fever, rigidity, altered mental status, and autonomic instability. Its presentation overlaps with malignant catatonia, complicating the diagnosis. Atypical courses are increasingly recognised with second-generation depot antipsychotics, where delayed clearance may prolong illness.

**Objectives:**

We present a diagnostically complex case of NMS with catatonic features, describe therapeutic dilemmas, and highlight the importance of multidisciplinary collaboration and electroconvulsive therapy (ECT).

**Methods:**

Anonymised inpatient records, laboratory data, and multidisciplinary discussions were reviewed against criteria for NMS and catatonia.

**Results:**

**(Case Report)** A 50-year-old woman with Schizophrenia and Intellectual Disability, stable on oral Aripiprazole, relapsed after psychosocial stressors and non-adherence. Following depot Aripiprazole and oral Olanzapine, she developed mutism, poor intake, agitation, fever, autonomic instability, elevated CK, and fluctuating rigidity. Catatonic signs such as waxy flexibility, verbigeration and psychological pillow soon emerged. Organic causes were excluded and NMS diagnosed. Antipsychotics were stopped; supportive care, IV Lorazepam, Dantrolene, and oral Bromocriptine were given. Despite this, she remained stuporous. Complications included DVTs (Deep vein thrombosis), rectal bleeding, and lorazepam-induced hypotension, requiring high-dependency care. ECT was delayed due to instability but once started, produced steady improvement. By the ninth ECT, she resumed independent eating and mobility. Low-dose Aripiprazole was cautiously reintroduced.

**Discussions:**

This case illustrates the diagnostic complexity of atypical NMS overlapping with malignant catatonia. Rigidity emerged late, delaying recognition, while catatonic features complicated diagnosis. Lorazepam escalation gave only transient benefits. ECT, though considered early, was postponed due to gastrointestinal bleeding, hypotension, and autonomic instability; when delivered, it was highly effective, raising the question of whether earlier initiation might have reduced morbidity. Additional challenges included depot Aripiprazole prolonging severity, national Dantrolene shortages, and family distress over treatment limitations. Recovery was achieved through sustained multidisciplinary collaboration, underlining the need for flexible diagnostic frameworks and timely access to ECT.

**Conclusions:**

Atypical NMS may mimic malignant catatonia, delaying recognition and treatment. Depot antipsychotics can prolong severity, while drug shortages complicate management. ECT ultimately yielded recovery after a protracted course, achieved only through multidisciplinary care. This case underscores the resilience of patients and the importance of system preparedness for rare but life-threatening neuropsychiatric emergencies.

**Disclosure of Interest:**

None Declared

